# The impact of mentorship program on the level of anxiety and pre-internship exam scores among Iranian senior nursing students

**DOI:** 10.1186/s12912-024-01835-x

**Published:** 2024-03-14

**Authors:** Farzaneh Arab, Maryam Saeedi

**Affiliations:** Nursing department, Saveh University of Medical Sciences, Saveh, Iran

**Keywords:** Mentor, Students, Nursing, Test anxiety

## Abstract

**Background:**

Mentorship involves a voluntary, collaborative, and non-hierarchical relationship where an experienced individual shares knowledge with a less-experienced individual. This study aimed to evaluate the effects of a mentorship program on anxiety levels and pre-internship exam scores among senior nursing students.

**Methods:**

This quasi-experimental research was conducted on 37 nursing students in the sixth semester of the School of Medical Sciences in Saveh in the year 2023. Participants were selected based on initial criteria using a census method and were then randomly assigned to two groups: the control group (19 participants) and the intervention group (17 participants). The mentoring program for the intervention group was implemented one month before the pre-internship exam and consisted of six sessions, each lasting two hours, over two weeks (three sessions per week). Data collection tools included a demographic questionnaire, the Sarason Anxiety Questionnaire, and pre-internship exam scores. SPSS software version 23 was utilized for data analysis.

**Results:**

The mean anxiety exam scores in the two control and intervention groups did not exhibit a statistically significant difference before the intervention (*P* = 0.34). However, the mean anxiety exam score of the intervention group students after the intervention (5.89 ± 15.11) was significantly lower than that of the control group students (7.04 ± 21.42) (*P* = 0.007). Additionally, the results showed that the mean anxiety exam scores of the intervention group students before (5.77 ± 17.53) and after the intervention (5.89 ± 15.11) had a statistically significant difference (*P* = 0.013). Furthermore, the mean pre-internship exam scores of the intervention group students (1.71 ± 17.72) were significantly higher than those of the control group students (1.15 ± 16.46) (*P* = 0.014).

**Conclusion:**

The mentorship program resulted in a reduction of exam anxiety in nursing students and improved their performance in the pre-internship exam to the extent that the exam scores of the participating students were higher than those of other students.

## Background

Assessment is an integral and crucial part of nursing education. One of the methods for assessing clinical skills and the performance of nursing students is the Objective Structured Clinical Examination, commonly known as the OSCE (Objective Structured Clinical Examination) [1]. In the OSCE, theory and practice are integrated, allowing for the evaluation of learners’ skills in all three cognitive, affective, and psychomotor domains. In general, the OSCE is designed to assess the clinical skills and competencies of students for real-world clinical responsibilities [2].

One assessment method that requires students to navigate various stations and be evaluated on different topics is the OSCE. Each station encompasses a clinical scenario where students are required to perform standardized patient interviews or solve a clinical problem. The performance of each student is assessed by an evaluator using a structured checklist designed for each station. While every assessment method has its advantages and disadvantages, the OSCE is known for its consistency among participants and its ability to simulate real-world conditions. However, it can be time-consuming, resource-intensive, and anxiety-inducing [3].

Regarding anxiety, studies have shown mixed results regarding the anxiety levels associated with the OSCE. Some studies suggest that the OSCE is less stressful for students and leads to greater satisfaction compared to traditional assessment methods [4]. However, other studies indicate that while exam-related anxiety is more pronounced with the OSCE, it has a minimal correlation with student performance [5].

Exam anxiety is defined as a state of discomfort, complexity, and ambiguity that occurs during the evaluation of situations based on progress. Nursing students, whether at the undergraduate or graduate level, are at a higher risk of experiencing stress and anxiety compared to students in other fields. The competitive nature of nursing education and the complexities of clinical and educational experiences are common sources of stress and anxiety among nursing students, which can lead to physical and psychological distress [6].

One of the innovative approaches that has gained significant attention in recent years in nursing education and has been shown to enhance clinical competencies is mentorship [7]. Mentorship is a form of voluntary, supportive, collaborative, and non-hierarchical relationship in which an experienced individual (mentor) imparts knowledge and experience to a less-experienced individual (mentee) and serves as a role model [8].

Studies suggest that mentorship contributes to improvements in communication skills, stress reduction, increased self-confidence, knowledge acquisition, enhanced sense of responsibility, and improved problem-solving. Ultimately, these benefits elevate the standards of patient care and leadership skills among students [9–11].

Several studies have emphasized the positive impact of the mentorship model on reducing stress for nursing students in clinical settings. This model facilitates adaptation to university life and the nursing profession, increases self-confidence and self-awareness, and promotes effective stress-coping mechanisms. Some researchers, including Bagherieh, Mikkonen, and Demir, have recommended mentorship as a suitable method in nursing education [12–14].

Given the importance of teaching and learning clinical skills, evaluating the scientific and practical skills of nursing students before entering clinical rotations through an objective structured examination is essential. On the one hand, the unfamiliarity of nursing students with OSCE procedures can lead to anxiety and hinder their performance in scientific and practical skills. Considering that the impact of mentorship before the pre-internship exam through the OSCE has not been previously studied in nursing students, we decided to conduct a study to determine the effect of a mentorship program on the level of anxiety and pre-internship exam scores in senior nursing students.

## Methods

### Study design

The present study was a quasi-experimental two-group (control and intervention) research conducted in 2023 to investigate the effect of a mentoring program on anxiety and pre-internship exam scores of sixth-semester nursing students at the Saveh University of Medical Sciences. The inclusion criteria for this study were: sixth-semester nursing students eligible to choose clinical internships, who had not participated in any objective-structured clinical exams in previous semesters. The exclusion criteria included incomplete questionnaires, absence from the pre-internship exam, and non-attendance of intervention group students in training sessions.

### Participants and setting

The research was conducted at Saveh University of Medical Sciences, situated in the central state of Iran. The university offers programs in medical, nursing, and various paramedical fields. On average, the institution admits 35 to 50 nursing students annually through a nationwide entrance examination.

To conduct this research, we selected all sixth-semester nursing students eligible for pre-internship exams in the first half of 2023 through a census, adhering to entry criteria. After explaining the research objectives and obtaining informed written consent, they participated in the study and were randomly divided into two groups: the intervention (mentoring) group and the control group. The mentoring program for the intervention group started one month before the pre-internship exam and was conducted over six two-hour sessions, spanning two weeks (3 sessions per week). In this program, a mentor who was a faculty member with a master’s degree in nursing and five nursing students who had previously passed the pre-internship exam and entered clinical internships provided guidance to the intervention group students regarding readiness for the pre-internship exam. Each session was dedicated to a specific topic, and at the end of each session, students’ questions were answered. The topics of each session are presented in Table 1.

**Table 1:** The topics of each session in the mentorship program

Session	Topics	Length of each session
1	Introduction to the pre-internship exam, exam titles, study resources, and stress management techniques.	2 h
2	Basic skills and sterile procedures.	2 h
3	Medications and intravenous therapy.	2 h
4	Nursing process and patient education.	2 h
5	Emergencies and specialized care.	2 h
6	Key points in maternal and child health.	2 h

### Measurements

Data collection tools included a demographic questionnaire, the Sarason Anxiety Questionnaire, and pre-internship exam scores.

The Sarason Exam Anxiety Questionnaire [15] consists of 37 items that are answered with “yes” or “no.” This questionnaire allows individuals to self-report their psychological state and physiological experiences before and after the exam. Higher scores on this questionnaire indicate higher exam anxiety. Scores of 12 and below represent mild anxiety, scores between 13 and 20 represent moderate anxiety, and scores of 21 and above indicate severe anxiety. The validity and reliability of this questionnaire have been confirmed in multiple studies, with a Cronbach’s alpha coefficient of 0.88, internal consistency of 95%, and criterion validity of 0.72 [16]. Cronbach’s alpha coefficient for the questionnaire in this study was 0.79.

Before implementing the mentoring program, both groups of students underwent a pre-test (using the Sarason Exam Anxiety Questionnaire). On the day of the pre-internship exam, and before conducting the exam, the anxiety of both intervention and control groups was re-evaluated, and the anxiety scores of both groups were compared using the Sarason Exam Anxiety Questionnaire (post-test). At the end of the pre-internship exam, the exam scores of both control and intervention groups were recorded and compared.

### Data analysis

For data analysis, SPSS version 23 and descriptive statistical methods, including calculating means and frequencies to describe the data, as well as analytical statistical methods, including paired t-tests, independent t-tests, Mann-Whitney, and Fisher’s tests, were used to compare anxiety scores between intervention and control groups and to compare exam scores between the control and intervention groups.

### Ethics consideration

The study protocol was approved by the Ethics Committee of Saveh University of Medical Sciences (Code: IR.SAVEHUMS.REC.1401.031) and complied with the requirements of the Helsinki Declaration. Informed consent was obtained from all participants to voluntarily participate in the research. Participants were informed that their participation in the study was optional and that they had the right to withdraw if they did not wish to continue their participation. All information of participants was analyzed and reported confidentially. The rights of the authors of the texts used in the research were respected, with proper citation of the sources.

## Results

In this study, a total of 37 sixth-semester nursing students participated, with 17 students in the intervention group and 19 students in the control group, randomly assigned. The Kolmogorov-Smirnov test was used to assess the normality of quantitative data, which showed that variables such as age and the previous semester’s grade point average (GPA) did not follow a normal distribution (*P* < 0.05). Therefore, non-parametric tests were used for the statistical analysis of these data. Variables such as pre-internship exam scores and pre- and post-intervention anxiety scores were normally distributed (*P* > 0.05), and parametric tests were used for statistical analysis of these data. The demographic information of research participants, grouped by intervention and control, is presented in Table 2. Based on the results, there were no significant differences between the intervention and control groups in terms of demographic variables.

**Table 2:** Comparison of demographic information of samples in intervention and control groups

variables	Grouping	Intervention	Control	total number	Significance Level
		number (percentage)	number (percentage)		
gender	girl	(25)9	(27.8)10	(52.8)19	0.62*
	boy	(22.2)8	(25)9	(47.2)17	
Job	Yes	(19.4)7	(19.4)7	(38.9)14	0.83*
	No	(27.8)10	(33.3)12	(61.1)22	
Age	Mean ± Standard Deviation	22.53 ± 3.53	21.89 ± 0.56		0.44**
The average of the previous semester	Mean ± Standard Deviation	16.35 ± 4.23	17.56 ± 1.14		0.26*

*Fisher’stest  ** Mann-Whitney test

Based on the results of this research, the average pre-intervention anxiety exam scores in the two control and intervention groups did not have a statistically significant difference (*P* = 0.34). However, after the intervention, a statistically significant difference was observed in the average anxiety exam scores between the control and intervention groups (*P* = 0.007). The average anxiety exam score of students in the intervention group after the intervention (5.89 ± 15.11) was significantly lower than the average anxiety exam score of students in the control group (7.04 ± 21.42). Additionally, the findings showed that the average anxiety exam scores of students in the intervention group before (5.77 ± 17.53) and after the intervention (5.89 ± 15.11) had a statistically significant difference (*P* = 0.013). This means that the anxiety exam scores of students in the intervention group significantly decreased after the intervention. However, such a result was not observed in the control group. These results indicate that the mentoring program significantly reduces the exam anxiety of nursing students (Table 3).

Furthermore, a comparison of the average pre-internship exam scores of students showed a statistically significant difference in the average pre-internship exam score between the intervention and control groups in the post-intervention period (*P* = 0.014). Specifically, the average pre-internship exam score of students in the intervention group (1.71 ± 17.72) was significantly higher than that of students in the control group (1.15 ± 16.46). In other words, the mentoring program led to better performance by nursing students in the pre-internship exam (Table 3).

**Table 3:** Comparison of mean and standard deviation of pre-internship test scores and students’ anxiety in intervention and control groups

	Anxiety score		Pre-internship test scores	Significance level
	Before	after	after	
group	Standard Deviation ± Mean	Standard Deviation ± Mean	Standard Deviation ± Mean	
intervention	17.53 ± 5.77	15.11 ± 5.89	17.72 ± 1.71	0.013*
Control	19.42 ± 6.01	21.42 ± 7.04	16.46 ± 1.154	0.073*
Significance level	**0.34	**0.007	0.014	

* Paired t-test

** Independent T-test

## Discussion

The results of the present study showed that the mentorship program led to a reduction in test anxiety among intervention group students compared to the control group. Additionally, the pre-internship exam scores in the mentorship group were higher than those in the non-mentorship group, indicating that the mentorship program resulted in improved performance among nursing students.

In line with the findings of the current research, the results of Demir and colleagues, who conducted a study to examine the effectiveness of a 14-week mentoring program on stress coping and locus of control among first-year nursing students, showed that mentoring guidance led to the improvement of problem-solving skills, adaptation to the university environment, self-awareness, self-confidence, and the development of positive relationships among first-year students with their mentors [13]. In our study, the implementation of the mentorship program one month before the pre-internship exam resulted in improved student performance in the exam and a reduction in student anxiety compared to the control group. Given these positive results, it is recommended to use mentoring programs as an additional tool to facilitate the adaptation of students to the university and nursing profession, as well as to help them cope with stress and internal control sources.

In accordance with the present research results, Mashalchi and colleagues also reported that the mentorship program had a positive and meaningful effect on the self-esteem, anxiety, and clinical skills of medical emergency nursing students. In general, mentorship is a student-centered approach and can be an effective participatory strategy to enhance student capabilities. Therefore, it is essential to develop a clinical education program that provides a path for clinical skill development [17]. In our study, the results showed that the mentorship program led to improved performance and pre-internship exam scores among intervention group students. Aligned with the findings of the present research, Jacobik and colleagues noted in their study that the nursing mentorship program resulted in a reduction of students’ anxiety and a boost in nurses’ self-efficacy [18]. In general, mentorship is a student-centered approach and it can be an effective participatory strategy to enhance student capabilities. Therefore, it is essential to develop a clinical education program that provides a path for clinical skill development.

In comparison to the results of the current research, Bahar and colleagues reported in their study that peer teaching had no significant impact on clinical skills learning and nursing student anxiety, but post-exam anxiety levels were lower in the experimental group than in the control group [19]. In our study, the level of anxiety in the intervention group was significantly lower than that in the control group. However, the scores and performance of students in the pre-internship exam were higher in the mentorship group than in the non-mentorship group, which was statistically significant. In our study, the mentorship program was conducted by a faculty member with a master’s degree in nursing and five nursing students who had previously passed the pre-internship exam. The control group of students did not participate in any training. However, in Bahar’s study, ten senior students volunteered to identify and train their peers. Training for the control group students was conducted by course instructors.

Studies indicate that students, both at the undergraduate and postgraduate levels, face elevated stress and anxiety owing to the competitive and intricate nature of nursing programs. Mentoring alleviates situational or short-term anxiety and enhances student participation and competence. The reduction of stress and anxiety by a mentor can foster self-confidence and positive outcomes among nursing students [6].

Derived from the research findings, mentorship, as a student-centered and collaborative educational approach, has the potential to enhance skills, reduce anxiety, and improve clinical performance in nursing students. Considering the significance of clinical education and assessment in nursing and the necessity for nursing educators to contribute to the enhancement of clinical education quality through the adoption of innovative teaching methods, the results of this research can serve as a guiding resource. Based on the findings of this research, it is suggested that the implementation of mentorship in nursing education can result in more effective and higher-quality outcomes. Implementing the mentorship method, while being easy to use and cost-effective, can diminish the gap between university and hospital settings. Furthermore, the outcomes of this study prompt researchers to explore the efficacy of mentorship training programs in additional clinical examinations.

## Limitations

The limitations of this study include the small sample size and the selection of students from only one university, which can limit the generalizability of the findings.

## Conclusion

This study demonstrated that the mentorship program led to a reduction in anxiety and an improvement in the scores and performance of senior nursing students in the pre-internship exam. Since nursing is a complex field and both students and nurses face stressful and demanding conditions, it is recommended to use mentoring programs as an additional tool to enhance the scientific and practical skills of nursing students, especially in clinical skills in clinical training units and other fields. Experienced nurses should also be used as mentors alongside recent nursing graduates at the beginning of their nursing careers to better manage anxiety and improve performance.

## Data Availability

The datasets analyzed during the current study are available from the corresponding author upon reasonable request. The data are not publicly available due to privacy and ethical restrictions.

## Abbreviations

OSCE: Objective Structured Clinical Examination
